# 

**Published:** 1882-05

**Authors:** 


### CONTENTS:

ART. I.—An Investigation into the Effects of Organisms Upon the Teeth and Alveolar Portions of the Jaw.............1

ART. II.—Characteristics of Saliva in Syphilitics. By Dr. A. W Harlan.............8

ART. III.—Secondary Dentine. By John G. Harper D. D S.............17

ART. IV.—On the Causes and Treatment of Tartar By O. G. Duncan.............23

ART. V.—How to Treat Alveolar Abscess. By L. D. Carpenter.............29

ART. VI.—Treatment of the Mouth During the Eruption of the Permanent Teeth....33

ART. VII.—Spontaneous (?) Abrasion of the Teeth By Dr L. H King.............35

Texas State Dental Association Proceedings.............38

Pennsylvania State Dental Society.............40

### EDITORIAL, ETC.

The Dental Department of the University of Maryland.............41

Life Insurances.............43

Faculty Changes.............43

Southern Dental Association.............44

### MONTHLY SUMMARY.

Anatomical Anomalies.............45

Large Odontome Removed from the Lower Jaw.............46

A New Cure for Alveolar Abscess.............47

Communicability of Syphilis by Suckling.............47

Death from Carbolic Acid.............47

Dentistry as a Specialty.............48

Chloralated Tincture of Iodine.............48

